# Revision tracheal resection anastomosis for recurrent stenosis after airway surgeries: functional outcomes

**DOI:** 10.1007/s00405-025-09441-6

**Published:** 2025-05-20

**Authors:** Hoda Abd-Elmageed Mansour, Ahmed Musaad Abd-Elfattah, Elsharawy Kamal, Hisham Atef Ebada

**Affiliations:** Department of Otorhinolaryngology, Mansoura University, Mansoura, 35511 Egypt

**Keywords:** Tracheal, Cricotracheal, Resection, Anastomosis, Outcomes, Quality of life

## Abstract

**Objectives:**

To evaluate the surgical and functional outcomes as well as the quality of life among patients who underwent revision tracheal/cricotracheal resection anastomosis for recurrent stenosis after previous unsuccessful airway surgeries.

**Methods:**

This prospective study was conducted on 53 patients. Circumferential resection of the stenotic airway segment was done with primary end-to-end anastomosis. All surgeries were performed by the authors of this work with the same standardized surgical techniques. To decrease the anastomotic tension, suprahyoid release was performed for all patients and trachea-hyoid detensioning stitches were placed. Surgiflo was applied over the line of the anastomosis to enhance healing. Surgical and functional outcomes were evaluated.

**Results:**

Types of anastomosis were cricotracheal anastomosis (*n* = 18), thyrotracheal anastomosis (*n* = 24), and tracheo-tracheal anastomosis (*n* = 11) according to the remaining proximal and distal stumps. The overall decannulation rate was 92.5% (49 out of 53 patients). No major intraoperative complications were reported. Postoperative complications were reported in 13 patients (24.5%), in the form of restenosis (*n* = 7), granulation tissue formation at the site of anastomosis (*n* = 5), surgical emphysema / minor air leak through drains (*n* = 4), unilateral vocal fold paralysis (*n* = 2), wound seroma (*n* = 1). Regarding functional outcomes, dyspnea was considerably alleviated both at rest and during exercise, and most patients had satisfactory voice and swallowing related functions. The majority of patients reported adequate QOL.

**Conclusion:**

Revision tracheal/cricotracheal resection anastomosis presents significant surgical challenges. Nevertheless, by employing meticulous surgical techniques and implementing strategies to reduce anastomotic tension and enhance healing such as suprahyoid release, trachea-hyoid detensioning stitches, and the application of surgiflo, high success rates and satisfactory functional outcomes were achieved.

## Introduction

The surgical options for treatment of laryngotracheal stenosis include intraluminal procedures (dilatation, stent placement, and laser surgery), laryngo-tracheal reconstruction (LTR) using cartilage grafts, and tracheal or cricotracheal resection anastomosis [1, 2, 3].

Dilatation and laser surgery are safe and well-tolerated procedures, but they typically lead to only short-term symptom relief. Recurrence is common necessitating repeated procedures without a conclusive outcome [4, 5, 6, 7]. Although open procedures (LTR and tracheal/cricotracheal resection anastomosis) have high success rates [2, 1, 8, 9, 10], the incidence of restenosis after these surgeries is between 3 and 9.5% [8, 9, 10, 11, 12, 13].

Management of recurrent stenosis after previous airway surgery is challenging. Fibrosis resulting from the initial open surgery restricts the mobility of the remaining trachea. Furthermore, revision surgery is difficult due to unavoidable anastomotic tension because additional trachea is resected [14].

A successful outcome after airway surgery is usually defined in terms of surgical success, such as decannulation rates, recurrent stenosis, and the need for further interventions. Although functional outcomes such as breathing, voice and swallowing-related functions may be as important as surgical outcomes in defining success, they received little attention in the literature [15]. Additionally, patient reported outcomes and quality of life (QOL) are becoming increasingly applied for evaluation of treatment outcomes in many medical disciplines [16, 17].

Overall, few studies describe the functional outcome of patients after cricotracheal resections [18]. Laryngotracheal surgery proves effective in restoring breathing capacity while preserving vocal function. Even in cases of high-grade and complex airway stenosis, favorable functional outcomes can be achieved [19].

It is well established that the outcomes of revision airway surgeries are less satisfactory than primary surgeries [20, 21]. To the best of our knowledge, there are currently no studies in the literature that assess the functional outcomes of revision tracheal resection anastomosis. The aim of the current study was to evaluate the surgical and functional outcomes as well as the quality of life among patients who underwent revision tracheal/cricotracheal resection anastomosis for recurrent stenosis after previous unsuccessful airway surgeries.

## Methods

### Study design

Prospective cohort study.

### Setting

This study was conducted in the Otorhinolaryngology Department, Faculty of Medicine, Mansoura University, Egypt, over the past 5 years (June 2019– June 2024).

### Participants

Fifty-three patients who underwent tracheal/cricotracheal resection anastomosis for recurrent stenosis after previous failed airway surgeries were included in the current study. Informed written consents were obtained from all participants and the study was approved by the Mansoura Faculty of Medicine Institutional Research board (IRB: MD.19.12.262.R1).

### Surgical techniques

All patients (*n* = 53) underwent a clinical evaluation that involved a comprehensive history of their prior airway surgeries, as well as a trans-nasal flexible laryngoscopy to evaluate airway patency and vocal fold mobility. The grades and levels of stenosis were further evaluated under general anesthesia. The Myer Cotton grading system [22] was adopted and applied for grading of stenosis. In cases of grade four stenosis (complete airway obliteration), where the endoscope could not pass beyond the obstruction, evaluation of the distal airway was conducted through the existing tracheostoma to rule out double stenosis and to measure the length of the distal trachea in individuals who underwent previous tracheal resections.

The authors of the current work adopted the same surgical techniques that were described in their previous reports [20, 21, 23, 24]. Suprahyoid release was the standard procedure, and it was carried out in all patients (*n* = 53). Tailored suprahyoid release was performed according to the length of resection. A limited suprahyoid release was performed when the resected segment is short (less than 3 rings). This entails cutting the suprahyoid muscles attached to the body of the hyoid (mylohyoid and geniohyoid), and the stylohyoid tendon attached to the lesser horn, with preservation of the digastric slings on either side. On the other hand, in longer resections (more than 3 rings), full suprahyoid is performed. The release is extended more laterally to both greater horns with cutting of the digastric slings. In instances where suprahyoid release was performed during previous tracheal resection surgeries, a revision of this release was undertaken. This was achieved by division of the fibrosis located at the site of the previous release, just above the hyoid bone.

Primary end-to-end anastomosis was performed. After completion of the anastomosis, Surgiflo^®^ (Ethicon, Somerville, USA) was spread over the entire surgical area with particular emphasis on the airway anastomosis site, to ensure proper hemostasis and support the healing process.

Additionally, to decrease anastomotic tension, tracheo-hyoid de-tensioning stitches were performed in all surgeries. One or two 2/0 Vicryl stitches were performed, from the hyoid bone to the distal tracheal segment, one or 2 rings below the anastomosis.

Single stage surgery was the method of choice and was usually performed whenever possible. It involves immediate extubation upon completion of the procedure. This approach was utilized in 45 out of 53 patients. In contrast, double stage surgeries involve performing a tracheotomy below the anastomosis with planned later decannulation. This option was typically selected for young children under the age of 2 years, or patients with significant co-morbidities such as respiratory and neurological conditions with increased risk of post operative aspiration and chest infection. Double stage approach helps secure the airway and minimizes the risk of early postoperative complications, such as choking, aspiration, and surgical emphysema. Decannulation was performed 2 to 3 weeks after adequate healing of the anastomosis. In this study, double stage surgery was carried out on 8 patients (5 adults and 3 children).

At our institute, the chin to chest suture is not preferred, and it was not applied to any patients in this work. Patients and their caregivers were advised to restrict neck movements and avoid neck extension during the initial postoperative period of 2 to 3 weeks.

Following extubation, patients were admitted to the intensive care unit for monitoring for a duration of 24 h. On the next day, they were moved to the ward. Systemic antibiotics were prescribed along with short course of corticosteroids, mucolytics and proton pump inhibitors. Drains were removed on the seventh day after surgery, after which the patients were discharged. Follow-up appointments were scheduled weekly for the first month and then monthly for the next six months.

### Outcomes

The QOL and functional outcomes (breathing, voice, swallowing) were assessed 3 months after surgery. QOL was assessed by the EuroQol five dimensions questionnaire (EQ-5D) [25], evaluating five aspects of perceived health problems, including mobility, self-care, usual activities, pain or discomfort, and anxiety or depression. The EuroQol visual analogue scale (EQ-VAS, 0–10) was applied: 0 means the worst health imaginable and 10 means the best health imaginable. A total score (ranging from 0 to 50) was recorded.

Breathing was evaluated using the questionnaire that was adopted by Timman et al. [15]. The questionnaire included five items: dyspnea at rest, dyspnea during exercise, coughing, wheezing, and stridor. These items were assessed with a Visual Analog Scale (VAS) ranging from 0 to 10, where the left end was labeled ‘no complaints’ and the right end was labeled ‘severe complaints.’ A total score (ranging from 0 to 50) was recorded.

Voice was evaluated by the voice handicap index (VHI) [26]. The VHI is a 30-item self-administered, validated questionnaire, designed to assess the impact of voice impairment in three different domains: functional, emotional and physical. A 4-point interval score from “never” (0 points) to “always” (4 points) was used to indicate the frequency of complaints. These scores were combined to assign a total score (ranging from 0 to 120), with higher scores indicating more severe voice impairment.

Swallowing was evaluated by the dysphagia handicap index (DHI) [27]. DHI is a patient-administered 25-item questionnaire that measures the handicapping effect of dysphagia on the emotional, functional, and physical aspects of the patient’s life. The DHI has 9 questions in the functional subscale, 9 questions in the physical subscale, and 7 questions in the Emotional subscale. For each question there are three choices for the answer, Never, Sometimes, and Always, with a suggested scoring of 0, 2, and 4, respectively, making the range of the total DHI score 0-100.

Additionally, analysis of the preoperative and postoperative factors that may have an impact on the surgical and functional outcomes was performed. These factors included: age, previous airway procedures, level of stenosis, Myer-Cotton grade of stenosis [22], length of resection, type of anastomosis and whether the procedure is single or double stage.

### Statistical analysis and data interpretation

Data analysis was conducted using SPSS software, version 26 (SPSS Inc., PASW statistics for Windows version 26, Chicago: SPSS Inc.). Qualitative data were summarized using counts and percentages. For quantitative data, non-normally distributed data were represented with median values (along with minimum and maximum) while normally distributed data were described using mean ± standard deviation, following normality assessment with the Kolmogorov-Smirnov test. The significance level for the results was set at 0.05. Comparisons of qualitative data between groups were made using the Chi-Square test, Fisher’s exact test, or Monte Carlo tests as appropriate. The Kruskal-Wallis test and Mann-Whitney U test were utilized to compare two or more non-normally distributed groups, respectively. To evaluate the strength and direction of a linear relationship between two continuous or ordinal variables that are not normally distributed, the Spearman’s rank-order correlation was employed. Binary logistic regression was applied to examine the impact of a combination of more than two independent variables on a binary outcome using the Enter method. Additionally, multiple linear regression analysis was conducted to identify predictors of continuous outcomes that are normally distributed, including the calculation of R².

## Results

### Characteristics of the study population

This study included 53 patients (Table 1) with recurrent stenosis after previous airway procedures. Twelve patients had undergone the previous procedures in our center (dilatation in 10 patients, and tracheal resection in 2), while 41 patients were referred from other institutions to our tertiary referral center. Previous interventions included endoscopic/laser procedures (*n* = 24), tracheal/cricotracheal resections (*n* = 19) and laryngotracheal reconstruction (*n* = 10).

**Table 1:** Descriptive statistics of all studied parameters

	*N* = 53	%
Age / years Mean ± SD (min-max)	22.96 ± 13.42 (1–65)	
Pediatric	14	26.4
Adult	39	73.6
Level of stenosis		
Trachea	19	35.8
Subglottic	18	34.0
Mixed	16	30.2
Grade		
II	12	22.6
III	29	54.7
IV	12	22.6
Previous airway procedure		
Tracheal/cricotracheal resection	19	35.8
Laryngotracheal reconstruction	10	18.9
Dilatation / laser surgery	24	45.3
Operation		
Single stage	45	84.9
Double stage	8	15.1
Anastomosis		
Crico-tracheal	18	34.0
Thyro-tracheal	24	45.3
Trachea-tracheal	11	20.8
Resected segment length (cm)		
Median (min-max)	2.25(1–4)	
Complications	13	24.5
Quality of life score		
Median (min-max)	44(10–50)	
Breathing score		
Median (min-max)	45(11–49)	
Voice handicapping index		
Median (min-max)	15(0–78)	
Dysphagia handicap index		
Median (min-max)	4(0–80)	
Overall success		
Success	49	92.5
Failure	4	7.5

Age of included patients ranged from 1 to 65 years (mean 22.96). Thirty-nine were adults, while 14 were children (below 18 years). The grade of stenosis according to the Myer-Cotton grading system was grade II (*n* = 12), grade III (*n* = 29) and grade IV (*n* = 12).

### Surgical outcomes

Re-anastomosis was performed as cricotracheal anastomosis (*n* = 18), thyrotracheal anastomosis (*n* = 24), and tracheo-tracheal anastomosis (*n* = 11) according to the remaining proximal and distal stumps. The number of resected tracheal rings ranged from 1 to 4 rings (median 2.25). No intraoperative complications were reported in the current series. Postoperative complications were reported in 13 patients (24.5%), in the form of restenosis (*n* = 7), granulation tissue formation at the site of anastomosis (*n* = 5), surgical emphysema / minor air leak through drains (*n* = 4), unilateral vocal fold paralysis (*n* = 2), wound seroma (*n* = 1).

Restenosis was managed by repeated endoscopic dilatations. Successful outcome was obtained in 3 out of 7 patients, however, it was not successful in 4 patients where significant stenosis was observed, and those patients were tracheotomized and considered as surgical failure. Granulation tissue was managed by repeated excision with topical application of mitomycin. The patient with wound seroma required incision and drainage. Other complications (minor emphysema, air leak, and unilateral vocal fold paralysis) were successfully managed with conservative measures.

**Fig. 1:**
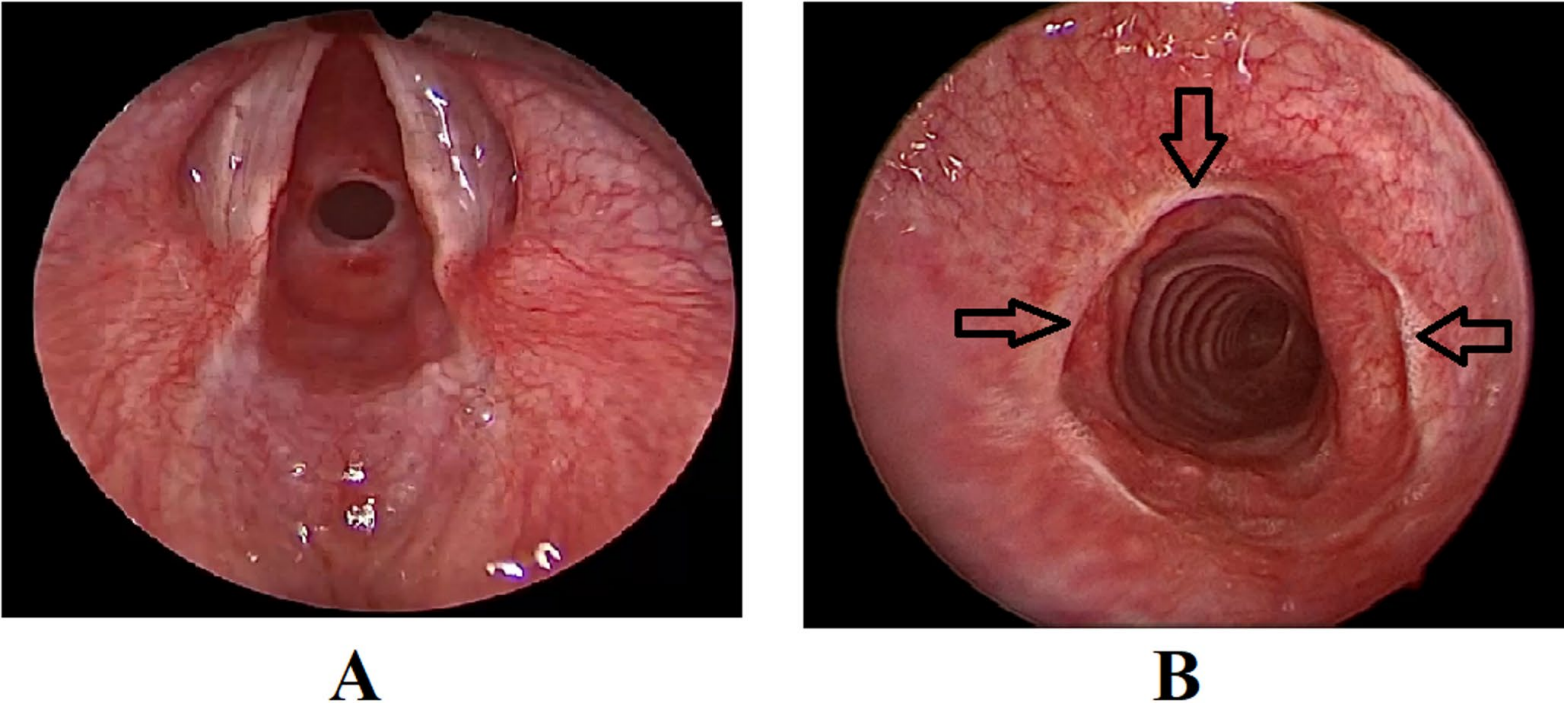
**A**: Endoscopic preoperative view of subglottic stenosis. Note the circumferential narrowing of the airway lumen. **B**: Postoperative successful outcome after tracheal resection anastomosis. Adequate healing is achieved with patent airway. Arrows point to the line of healing of the anastomosis

Successful decannulation was achieved in 49 out of 53 patients (92.5%) (Fig. 1). The follow up period ranged from 6 to 66 months (mean 32 months). No further airway interventions were indicated for those 49 patients in the follow up period, and they returned to their usual daily activities.

### Functional outcomes

Regarding the QOL and functional outcomes, the median QOL scores were 44 (10–50), the median breathing scores were 45(11–49), the median VHI scores were 15(0–78), and lastly the median DHI scores were 4(0–80). At the end of follow up period, dyspnea was considerably alleviated both at rest and during exercise, and most patients had satisfactory voice and swallowing related functions. The majority of patients reported adequate QOL.

By analyzing the possible impact of preoperative and postoperative factors on the outcomes (Tables 2, 3, 4 and 5), the Myer-Cotton grade of stenosis had a statistically significant impact on the rate of decannulation and the incidence of complications (*p* = 0.031 and 0.049, respectively). Similarly, previous airway intervention significantly impacted the decannulation rates and the incidence of complications (*p* = 0.009 and 0.002, respectively).

**Table 2:** Relation between risk factors and incidence of complications and overall success among studied cases

	Complications			Overall success (decannulation)		
	No *N* = 40	Yes *N* = 13	Test of significance	Success *N* = 49	Failure *N* = 4	Test of significance
Age / years	24.25 ± 13.62	19.0 ± 12.44	t = 1.23 *p* = 0.224	23.49 ± 13.66	16.5 ± 8.51	t = 1.0 *p* = 0.321
Pediatric	8(20)	6(46.2)	FET = 3.45	12(24.5)	2(50)	FET = 1.238
Adult	32(80)	7(53.8)	*P* = 0.08	37(75.5)	2(50)	*P* = 0.282
Level of stenosis						
Trachea	17(42.5)	2(15.4)	χ^2^=3.57	19(38.8)	0	χ^2 MC^ =4.53
Subglottic	13(32.5)	5(38.5)	*P* = 0.168	17(34.7)	1(25)	*P* = 0.104
Mixed	10(25.0)	6(46.2)		13(26.5)	3(75)	
Grade						
II	9(22.5)	3(23.1)	χ^2^=6.01	12(24.5)	0	χ^2 MC^ =6.92
III	25(62.5)	4(30.8)	*P* = 0.049*	28(57.1)	1(25)	*P* = 0.031*
IV	6(15)	6(46.2)		9(18.4)	3(75)	
Previous airway procedure						
Tracheal/cricotracheal resection	13(32.5)	6(46.2)	χ^2 MC^ =12.68	18(36.7)	1(25)	χ^2 MC^ =9.32
Laryngotracheal reconstruction	4(10.0)	6(46.2)	*P* = 0.002*	7(14.3)	3(75)	*P* = 0.009*
Dilatation / laser surgery	23(57.5)	1(7.7)		24(49)	0	
Operation						
Single stage	34(85)	11(84.6)	χ^2^=0.001	42(85.7)	3(75)	FET = 0.331
Double stage	6(15)	2(15.4)	*P* = 0.973	7(14.3)	1(25)	*P* = 0.491
Anastomosis						
Crico-tracheal	16(40)	2(15.4)	χ^2 MC^ =4.17	18(36.7)	0	χ^2MC^=5.23
Thyro-tracheal	15(37.5)	9(69.2)	*P* = 0.124	20(40.8)	4(100)	*P* = 0.073
Trachea-tracheal	9(22.5)	2(15.4)		11(22.4)	0	
Resected segment length (cm)	2.57 ± 0.78	2.65 ± 0.79	t = 0.342 *p* = 0.734	2.60 ± 0.79	2.44 ± 0.38	t = 0.406 *p* = 0.686

χ^2 MC^:Monte Carlo test, t: Student t test χ^2^=Chi-Square test, FET: Fisher exact test, *statistically significant

**Table 3:** Binary logistic regression for prediction of complications and overall success among studied cases

	Prediction of complications			Prediction of overall success		
	β	P value	Odds ratio (95%CI)	β	P value	Odds ratio (95%CI)
Grade of stenosis	1.56	0.029*	r 4.77(1.17-19.4203)	2.68	0.029*	r 14.57(1.31-162.08)
Previous airway procedure	-0.532	0.439	r 0.587(0.153–2.26)	0.845	0.507	r 2.33(0.192–28.21)

β: regression coefficient, *statistically significant, r: reference group

**Table 4:** Relation between risk factors and quality of life and functional outcomes among studied cases

	Quality of life score		Breathing score		Voice handicap index		Dysphagia handicap index	
	scores median (range)	Test of significance	scores median (range)	Test of significance	scores median (range)	Test of significance	scores median (range)	Test of significance
Age / years	*r* = 0.129	*p* = 0.357	*r*=-0.037	*p* = 0.793	*r*=-0.094	*p* = 0.505	*r*=-0.087	*p* = 0.535
Pediatric	36(18–49)	Z = 1.64	40(13–49)	Z = 0.142	21.5(0–78)	Z = 0.615	5(2–60)	Z = 0.238
Adult	44(10–50)	*P* = 0.101	45(11–48)	*P* = 0.887	15.0(0–68)	*P* = 0.539	4(0–80)	*P* = 0.812
Level of stenosis								
Trachea	44(10–50)	KW = 1.11	45(11–49)	KW = 2.31	0(0–30)	KW = 21.81	4(0–46)	KW = 4.56
Subglottic	44(20–50)	*P* = 0.575	45(18–49)	*P* = 0.316	21(0–74)	*P* = 0.001*	6 (2–60)	*P* = 0.102
Mixed	41(18–48)		39(11–48)		32(0–78)		6(2–80)	
Grade							4(0–80)	KW = 0.514
II	45(18–48)	KW = 0.229	45(11–48)	KW = 0.505	7(0–39)	KW = 1.38	4(2–60)	*P* = 0.773`
III	44(10–50)	*P* = 0.892	41(11–49)	*P* = 0.777	15(0–74)	*P* = 0.502	5(2–46)	
IV	41(10–50)		45(12–49)		23(0–78)			
Previous airway procedure								
Tracheal/cricotracheal resection	44(18–50)	KW = 1.19	45(13–48)	KW = 3.01	23(0–68)	KW = 32.37	4(2–60)	KW = 11.24
Laryngotracheal reconstruction	38(20–48)	*P* = 0.552	39(11–48)	*P* = 0.222	39(22–78)	*P* = 0.001*	9(2–80)	*P* = 0.004*
Dilatation / laser surgery	44(10–50)		0(0–25)		0(0–25)		4(0–26)	
Operation								
Single stage	44(10–50)	Z = 0.275	45(11–49)	Z = 0.675	15(0–73)	Z = 0.462	4(0–80)	Z = 0.434
Double stage	43(30–46)	*P* = 0.784	43(38–49)	*P* = 0.500	18(0–78)	*P* = 0.644	5(2–60)	*P* = 0.665
Anastomosis								
Crico-tracheal	45(20–50)	KW = 0.663	45(17–49)	KW = 1.02	0(0–74)	KW = 23.44	3(0–60)	KW = 7.34
Thyro-tracheal	43(18–50)	*P* = 0.718	45(11–48)	*P* = 0.602	26(0–78)	*P* = 0.001*	6(2–80)	*P* = 0.025*
Trachea-tracheal	40(10–50)		41(11–49)		0(0–16)		4(2–46)	
Resected segment length (cm)	*r* = 0.160	*p* = 0.254	*r* = 0.133	*p* = 0.341	*r*=-0.174	*p* = 0.214	*r* = 0.134	*p* = 0.340

Z: Mann Whitney U test, KW: Kruskal Wallis test, r:Spearman correlation

**Table 5:** Predictors of voice handicap index and dysphagia handicap index among studied cases

		Predictors of voice handicap index						Predictors of dysphagia handicap index					
Model		Unstandardized Coefficients		t	P value	95.0% Confidence Interval for B		Unstandardized Coefficients		t	P value	95.0% Confidence Interval for B	
		β	Std. Error			Lower Bound	Upper Bound	β	Std. Error			Lower Bound	Upper Bound
1	(Constant)	11.982	13.197	0.908	0.368	-14.539	38.502	3.331	13.823	0.241	0.811	-24.448	31.109
	Level of stenosis	1.502	3.432	0.438	0.663	-5.394	8.398	-4.774	2.766	-1.726	0.091	-10.333	0.784
	Previous airway procedure	-4.510	3.098	-1.456	0.152	-10.736	1.715	8.545	7.273	1.175	0.246	-6.071	23.162
	Type of anastomosis	3.322	3.458	0.961	0.341	-3.627	10.271	4.547	3.598	1.264	0.212	-2.683	11.777

β: Beta regression coefficient. Std.: standard

The voice related outcomes (VHI) were significantly affected by the level of stenosis (*p* = 0.001). Similarly, voice (VHI) and swallowing related outcomes (DHI) were significantly affected by the type of anastomosis (*p* = 0.001 and 0.025, respectively). Lastly, previous airway interventions had a significant impact on the voice and swallowing related outcomes (*p* = 0.001 and 0.004, respectively).

## Discussion

Few studies collected detailed information on functional outcomes after primary airway surgeries [15, 28, 29, 30], although these outcomes should be incorporated into the definition of treatment success. These studies demonstrated that laryngotracheal resection and anastomosis is safe and provides excellent surgical and functional outcomes with an improved quality of life. Additionally, functional outcomes were often assessed in previous literature using unstandardized subjective methods. In the current study, validated questionnaires [25, 26, 27] were applied to assess these outcomes.

Nauta et al. [29] described the functional outcomes of 34 patients who underwent tracheal resection and anastomosis, utilizing Visual Analogue Scales (VAS) for assessment. Their findings indicated a marked improvement in dyspnea scores following surgery, compared to baseline measurements. However, dysphagia scores remained largely unchanged. In terms of QOL, their results revealed that 29 patients (85%) reported improved QOL postoperatively, 2 patients (6%) noted no change, and 3 patients (9%) experienced a decline in their QOL relative to their condition prior to surgery. Similarly, Timman et al. [15] evaluated the functional outcomes of 30 patients who underwent single stage tracheal/cricotracheal resection anastomosis and reported significant improvement in breathing, voice, swallowing and QOL.

To our knowledge, the current work is the first study to investigate the functional outcomes and quality of life after revision tracheal resection anastomosis. Evermann et al. [30] reported that previous multiple endoscopic pretreatments lead to worse voice quality after tracheal resection anastomosis. The present study demonstrated that revision tracheal resection resulted in favorable surgical and functional outcomes, which are similar to those reported for primary surgeries in existing literature.

Tracheal/cricotracheal resection anastomosis after unsuccessful airway surgery is challenging [31]. In patients with a history of open airway surgeries, surgical dissection is difficult because of scarring and fibrosis resulting from the initial procedure. Fibrosis complicates the identification of anatomical landmarks, with increased risk of damaging critical structures such as the esophagus and recurrent laryngeal nerves. However, all revision surgeries were performed by our experienced airway surgery team with careful and meticulous dissection with no reported intraoperative complications.

Auchincloss and Wright [14] reported that scarring after prior surgeries limited the mobility of the trachea and lead to increased anastomotic tension. Evermann et al. [30] reported that previous failed endoscopic treatments usually result in more profound scarring compared to the initial presentation. A more cephalad extension of the scar is commonly observed due to additional damage caused by mechanical trauma/irritation or thermic tissue injury caused by laser. Patients may also have profound scaring outside the airway, complicating the dissection and mobilization [30].

The healing process following airway surgery significantly influences the outcome. Monnier [32] highlighted that achieving precise mucosa-to-mucosa alignment during tracheal anastomosis facilitates primary healing without the development of granulation tissue or fibrosis, ultimately leading to adequate healing without stenosis. Conversely, even minor dehiscence of the mucosa can trigger the formation of granulation tissue. This initiates the proliferative phase of healing, characterized by increased angiogenesis and fibroblast activity. Over the following weeks, the wound healing progresses into the remodeling phase which is characterized by development of mature scar tissue and airway restenosis [33, 34].

When performing revision tracheal resection after previous failed resection, anastomotic tension is unavoidable due to resection of more tracheal rings [35]. Several laryngotracheal release techniques have been proposed to reduce anastomotic tension. Monnier [36] noted that laryngeal release procedures can be performed and allow the removal of as many as 5 rings. Furthermore, he indicated that in pediatric cricotracheal resections, it is possible to resect up to 8 rings. Similarly, Taylor et al. [37] reported that up to 50% of the length of the trachea (8–10 rings) can be resected with full release maneuvers. Wright et al. [38] indicated that adult patients may undergo resection of as much as 50% of the trachea. In this study, the revision cricotracheal resection was successfully carried out with accepted anastomotic tension by utilizing a complete suprahyoid release.

Suprahyoid release was first described by Montgomery in 1974 [39]. This procedure is both safe and well tolerated, and it can be performed using the same cervical incision. For lower tracheal or carinal resections, mediastinal, hilar, and pericardial release techniques may be more effective, however, these techniques necessitate a separate thoracic incision. The release maneuver should be adequately performed if excess anastomotic tension is expected, especially during revision tracheal/cricotracheal resection [14]. The authors of the current work perform the suprahyoid release in a tailored manner according to the length of resection. Limited suprahyoid release is performed in short resections and full release in long resections.

Considering the aforementioned factors, it is unsurprising that in the current study, previous airway interventions showed a significant impact on outcomes. Patients who had undergone prior open airway procedures, such as laryngotracheal reconstruction or tracheal/cricotracheal resection, demonstrated a higher incidence of complications and an increased rate of decannulation failure. Similarly, Rea et al. [40] stated that previous airway interventions can increase the extent of tracheal injury and the length of stenosis. Previous tracheoplasty using costal cartilage or other methods is expected to increase damage and scarring in the cartilaginous framework of the airway. On the other hand, previous resection results in increased tension on anastomosis after further resection procedures.

In the current study, voice related outcomes were negatively affected by the level of stenosis (affection of the subglottic area) and the type of anastomosis (thyrotracheal anastomosis). A possible explanation is that when the subglottic area is involved, the cricoid arch was resected and thyrotracheal anastomosis was performed. In this type of anastomosis, the line of repair is closer to the under surface of the vocal folds compared to cricotracheal or thyrotracheal anastomosis. The resultant edema, scarring or stenosis in the subglottic region may negatively affect the mucosal waves and vibration of the vocal folds. Additionally, when cricoid resection is done the cricothyroid muscle, which overlies the anterior cricoid arch is detached. This muscle acts to increase the length and tension of the vocal folds. Consequently, loss of muscle function results in changes in the vocal fold tension and the pitch of voice [15].

Interestingly, Nakache et al. [41] reported lower success rates for patients with cricoid involvement (77%) compared to those without it (91.3%). Wright et al. [38] noted that cricoid involvement leads to a higher failure rate. They believed that performing laryngotracheal resection with anastomosis to the larynx is generally more challenging and appears to be less stable compared to tracheotracheal anastomosis. In the current study, decannulation rates were not affected by cricoid involvement, however the voice outcome was negatively affected.

Surgiflo was used at the conclusion of the procedure, applied to the surgical area and the anastomotic line. In their previous study [24], the authors of this work concluded that application of surgiflo significantly improved the success rates and decreased the incidence of anastomotic complications such as granulation tissue, surgical emphysema, and restenosis.

Surgiflo is an active flowable hemostatic matrix which contains gelatin and thrombin that perform via contact activation and improve the clotting cascade to stop bleeding. It has several advantages such as rapid hemostasis and precise application, as the gelatins in the flowable products fill and conform to the wound site shape, allowing versatility with delivery and effective tissue contact [42]. The authors of this study believed that application of Surgiflo might help fill any potential micro gaps in the anastomosis, thereby promoting healing and reducing the likelihood of anastomotic complications.

Trachea-hyoid detensioning stitches were applied to decrease the anastomotic tension, and consequently to decrease the incidence of anastomotic complications. Redmann et al. [43] described a similar procedure in which they placed internal suspension sutures using two 2 − 0 PDS through the two tracheal rings just distal to the anastomosis and then around the hyoid bone. They demonstrated that applying these suspension sutures alleviates the need for postoperative chin to chest sutures. Atallah et al. [44] described placement of a laryngosternopexy stitch between the thyroid lamina and the sternoclavicular ligament designed to take all of the tension off the anastomosis and to prevent inadvertent head extension.

Although the current work assessed the outcomes of revision tracheal/cricotracheal resection anastomosis, high surgical success rate (decannulation rate), and overall satisfactory functional outcomes and quality of life scores were achieved. This was similar to many previous reports which demonstrated high success rates [45, 46, 38] and satisfactory functional outcomes [15, 28, 29]. The incidence of complications in the current series was similar to the reported incidence in literature [44, 47]. The authors of this work believe that careful preoperative assessment, meticulous surgical techniques, and postoperative care are essential for achieving optimum results. Notably, the authors have implemented specific strategies to reduce anastomotic tension, including tailored suprahyoid release, tracheo-hyoid detensioning stitches, and the application of Surgiflo to promote healing. These measures are considered crucial in achieving such excellent outcomes.

This study has limitations, including a relatively small sample size, short term follow up, and its design as a single-center study. To evaluate the functional outcomes of these challenging surgical procedures more comprehensively, further multicenter studies involving a larger cohort of patients and long term follow up are necessary.

## Conclusion

Revision tracheal/cricotracheal resection anastomosis presents significant surgical challenges. Nevertheless, by employing meticulous surgical techniques and implementing strategies to reduce anastomotic tension and enhance healing such as suprahyoid release, trachea-hyoid detensioning stitches, and the application of surgiflo, high success rates and satisfactory functional outcomes were achieved.
